# Missouri

**Published:** 1895-05

**Authors:** 


### Missouri.

*Synopsis of House Bill No.* 667. *Passed House, March* 5, 1895.

Seetion 1 grants to State Board of Health the power to demand, through the Secretary of the State Board of Agriculture, the services of the State Veterinarian for the inspection of such infectious and contagious diseases as are transmissible to the human family, and in the examination of meats, milk, and foods.

Sect. 2 provides for any ten lawful freeholders through any officer of the courts calling for the presence of the State Veterinarian to examine into any outbreak of contagious or infectious diseases. Under Section 2 the necessary power to quarantine, disinfection of premises, erection of barriers of defense, is accorded the veterinarian. The power to order the destruction of diseased animals.

Sect. 3 provides for compensation when animals are destroyed through appraisers. Compensation for animals has been decreased as follows: Acute glanders from twenty-five dollars, not to exceed five dollars; subacute, from fifty to not exceed twenty-five, and chronic, from fifty to not exceed thirty dollars.

Sect. 4 provides for a cut in the State Veterinary Surgeon's salary from $2500 to $1800 yearly, with necesssry travelling and incidental expenses for each day employed.

We learn through State Veterinarian Turner that the bill making it a punishable offense for docking horses' tails, except by a qualified veterinarian, for remedying some physical defect, has been lost.

The Assembly bill revising the veterinary law of the State in which it establishes co-operation between the State Veterinary Sanitary Board and the State Board of Health, by which they will be enabled to do experimental work in regard to tuberculosis, has passed that body. The State Veterinarian's salary was cut down from $2500 to $1800 per year. This was accomplished by tacking on this as an amendment to the other bill, so that to defeat this change in salary would have carried with it the loss of the other important features of the bill.

A bill was introduced and referred to one of the committees to regulate the practice of veterinary science in the State. It is said to be very crude and lacked largely the indorsement of the profession of the State, and did not emerge from the committee.

### ARIZONA.

The salary of the State Veterinarian of Arizona has been cut down from $2000 to $1800.

